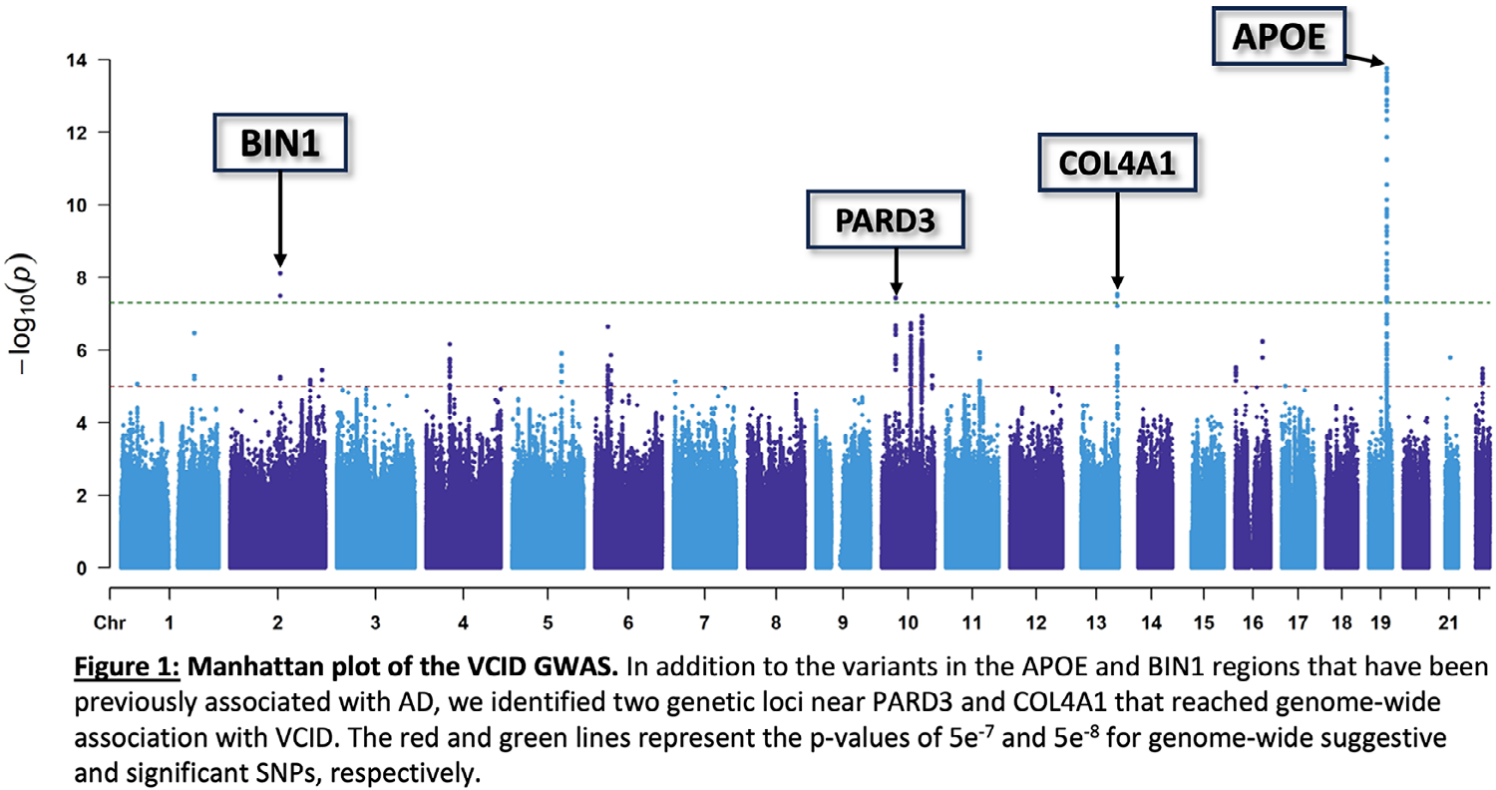# GIGAVCID: A Genome‐Wide Meta‐Analysis of 1.1 Million Individuals Identifies Novel Genetic Loci for Vascular Cognitive Impairment

**DOI:** 10.1002/alz70855_105753

**Published:** 2025-12-24

**Authors:** Bernard Fongang, Yannick Joel Wadop Ngouongo, Sami Heikkinen, Qiong Yang, Josh C. Bis, Bragi Walters, Kari Stefansson, Valborg Gudmundsdottir, Toshiharu Ninomiya, Jesper Qvist Thomassen, Xueqiu Jian, Nikhil Arora, Mohsen Sharifi Tabar, Muralidharan Sargurupremraj, Monica Goss, Ben Michael Brumpton, Bendik Winsvold, Elisa Amandine Moreno, Kumamoto Masaya, Yukihide Momozawa, Vilmundur Gudnason, Lenore J J. Launer, Oscar L Lopez, William T Longstreth, Myriam Fornage, Carole Dufouil, Ruth Frikke‐Schmidt, Arfan Ikram, Hreinn Stefansson, Stéphanie Debette, Mikko Hiltunen, Sudha Seshadri, Jean‐Charles Lambert, Patrick Gavin Kehoe, for CHARGE, deCODE Genetics/Amgen, CHB, UK Biobank, HUNT, JPSC AD, and EADB

**Affiliations:** ^1^ Glenn Biggs Institute for Alzheimer's & Neurodegenerative Diseases, The University of Texas Health Science Center at San Antonio, San Antonio, TX, USA; ^2^ Department of Population Health Sciences, The University of Texas Health Science Center at San Antonio, San Antonio, TX, USA; ^3^ Department of Biochemistry and Structural Biology, The University of Texas Health Science Center at San Antonio, San Antonio, TX, USA; ^4^ Glenn Biggs Institute for Neurodegenerative Diseases, University of Texas Health Science Center at San Antonio, San Antonio, TX, USA; ^5^ Institute of Biomedicine, University of Eastern Finland, Kuopio, Finland; ^6^ Boston University, Boston, MA, USA; ^7^ The Framingham Heart Study, Framingham, MA, USA; ^8^ Department of Biostatistics, Boston University School of Public Health, Boston, MA, USA; ^9^ University of Washington, Seattle, WA, USA; ^10^ deCODE Genetics/Amgen, Reykjavik, Iceland; ^11^ University of Iceland, Reykjavik, Iceland; ^12^ Graduate School of Medical Sciences, Kyushu University, Fukuoka, Japan; ^13^ Department of Clinical Biochemistry, Copenhagen University Hospital ‐ Rigshospitalet, Copenhagen, Denmark; ^14^ The University of Texas Health Science Center at San Antonio, San Antonio, TX, USA; ^15^ HUNT Center for Molecular and Clinical Epidemiology, Trondheim, Norway; ^16^ University of Texas Health Science Center at San Antonio, San Antonio, TX, USA; ^17^ Glenn Biggs Institute for Alzheimer's & Neurodegenerative Diseases, University of Texas Health Science Center, San Antonio, TX, USA; ^18^ Oslo University Hospital, Oslo, Norway; ^19^ Department of Epidemiology and Public Health, Graduate School of Medical Sciences, Kyushu University, Fukuoka, Japan; ^20^ Center for Cohort Studies, Graduate School of Medical Sciences, Kyushu University, Fukuoka, Japan; ^21^ Icelandic Heart Association, Kopavogur, Iceland; ^22^ Faculty of Medicine, University of Iceland, Reykjavik, Iceland; ^23^ Laboratory of Epidemiology and Population Sciences, National Institute on Aging, Baltimore, MD, USA; ^24^ National Institute on Aging, Baltimore, MD, USA; ^25^ University of Pittsburgh, Pittsburgh, PA, USA; ^26^ Human Genetics Center, School of Public Health, University of Texas Health Science Center, Houston, TX, USA; ^27^ University of Texas Health Science Center at Houston, Houston, TX, USA; ^28^ Univ. Bordeaux, INSERM, BPH, U1219, Bordeaux, France; ^29^ Pole de sante publique Centre Hospitalier Universitaire (CHU) de Bordeaux, Bordeaux, France; ^30^ Erasmus University Medical Center, Rotterdam, Zuid‐Holland, Netherlands; ^31^ University of Bordeaux, Bordeaux, France; ^32^ Bordeaux Population Health Research Center, Inserm U1219, University of Bordeaux, Bordeaux, France; ^33^ University of Eastern Finland, Kuopio, Finland; ^34^ Department of Neurology, Boston University School of Medicine, Boston, MA, USA; ^35^ Framingham Heart Study, Framingham, Boston, MA, USA; ^36^ Department of Neurology, University of Texas Health Sciences Center, San Antonio, TX, USA, San Antonio, TX, USA; ^37^ Univ. Lille, Inserm, CHU Lille, Institut Pasteur de Lille, U1167‐RID‐AGE, DISTALZ, Lille, France; ^38^ University of Bristol, Bristol, United Kingdom

## Abstract

**Background:**

Vascular cognitive impairment and dementia (VCID) is a leading cause of cognitive decline and often coexists with neurodegenerative pathologies such as Alzheimer's disease (AD). Despite its clinical significance, the genetic architecture of VCID remains poorly understood. Genome‐wide association studies (GWAS) have identified only *APOE* as significantly associated with VCID. The GIGAVCID project aims to conduct the largest GWAS of VCID and examine its genetic overlap with other traits. It is a collaborative effort between the Cohorts for Heart and Aging Research in Genomic Epidemiology (CHARGE) and the European Alzheimer's Disease DNA BioBank (EADB) consortia alongside major contributions from international cohorts (deCODE, HUNT, CHB, JPSC AD).

**Method:**

This study includes 16 cohorts with over 1.1 million individuals (74.8 ± 12.3 years, 56% female), including more than 15,000 VCID cases. Genotyping data were imputed using the TOPMed reference panel, and additional stratification was performed based on **APOE**ε4 genotype. Study‐specific analyses were adjusted for age, sex, and population structure. We then applied standardized quality control, data harmonization, and meta‐analysis using METAL, followed by conditional analyses and fine mapping.

**Result:**

We identified genome‐wide associations with VCID at the *APOE* and *BIN1* loci, both of which have been previously implicated in AD (Figure 1). Our novel findings include loci at or adjacent to the *PARD3* and *COL4A1* genes. *PARD3* plays a critical role in neurodevelopment by regulating neuronal differentiation, migration, and synaptic plasticity. *COL4A1* has been associated with cerebral small vessel disease (cSVD), a condition characterized by vascular fragility in the brain, increasing the risk of hemorrhagic strokes. Finally, we will present insights derived from pathway analyses and bioinformatic interrogation of the identified loci, providing a broader mechanistic understanding of their potential roles in VCID.

**Conclusion:**

Our findings reinforce the critical role of *APOE and BIN1* in VCID while identifying novel loci, including *COL4A1 and PARD3*. These discoveries suggest shared genetic mechanisms among VCID, AD, and cSVD. Our results provide valuable insights into the genetic architecture of VCID and underscore the need for further research to elucidate the biological pathways underlying vascular contributions to dementia. Future directions include functional annotation to validate and further characterize these genetic associations.